# Moroccan residents’ perceptions of the hospital learning environment measured with the French version of the Postgraduate Hospital Educational Environment Measure

**DOI:** 10.3352/jeehp.2020.17.4

**Published:** 2020-01-31

**Authors:** Hajar Berrani, Redouane Abouqal, Amal Thimou Izgua

**Affiliations:** 1 Pediatrics Department, Faculty of Medicine and Pharmacy, Mohammed V University-Rabat, Rabat, Morocco; 2 Laboratory of Biostatistics, Clinical Research and Epidemiology, Faculty of Medicine and Pharmacy, Mohammed V University-Rabat, Rabat, Morocco; Hallym University, Korea

**Keywords:** Residency, Morocco, Educational assessment, Educational Measurement

## Abstract

**Purpose:**

This study aimed to assess the educational environment of residents in Morocco and to compare residents’ perceptions depending on their specialty.

**Methods:**

We applied the French version of the Postgraduate Hospital Educational Environment Measure (PHEEM) to measure the educational environment at 6 hospitals in Rabat from January to June 2017. The internal reliability of the questionnaire was assessed using Cronbach’s α coefficient. Principal component analysis was conducted to assess the construct validity of the 3 subscales of the PHEEM questionnaire. Analysis of variance was performed to compare the mean scores of the overall PHEEM, its subscales, and each item among the 6 specialties.

**Results:**

Responses from 255 residents were included. The 40-item PHEEM questionnaire showed a high level of reliability, with a Cronbach’s α of 0.91. Principal component analysis of all 40 items suggested that 3 factors explained 48% of the variance, with better results for the teaching subscale. Moroccan residents perceived their educational environment as more positive than negative. There were significant differences in the overall and subscale scores among the 6 specialties.

**Conclusion:**

The French version of the PHEEM was confirmed to be a valid and reliable instrument in Morocco. Moroccan residents perceived their educational environment as more positive than negative, but room for improvement remained, with challenges including the poor infrastructure, the suboptimal quality of supervision, and inadequate teaching and work regulations.

## Introduction

In Morocco, physicians who graduate from medical schools enroll in residency programs that involve 3 to 5 years of training [1]. Thereafter, medical residents who complete residency training are certified as specialists. No previous studies in the literature have aimed to assess Moroccan residents’ perceptions of their educational environment. The clinical learning environment is an influential factor in work-based learning. This environment encompasses many important aspects, such as the quality of supervision, the quality of teachers, the facilities, and the atmosphere. Evaluation of the clinical learning environment may provide insights into the educational functioning of clinical departments. The Postgraduate Hospital Educational Environment Measure (PHEEM) is a 40-item inventory developed by Roff et al. [2] that covers a range of topics directly relevant to the educational climate of junior doctors; it has been reported to have high face validity both in the United Kingdom and in other international settings [3,4]. This inventory measures students’ perceptions of 3 domains (autonomy, teaching, and social support) during the hospital-based training period. The objectives of the present study were to assess the educational environment of residents in Morocco with the PHEEM and to compare perceptions of the educational environment among residents according to their specialty.

## Methods

### Ethics statement

The Rabat Ethics Committee for Biomedical Research, University Mohammed V, Faculty of Medicine (Rabat, Morocco) approved the protocol and consent procedure (IORG0006594). Written informed consent was obtained from the residents who participated.

### Study design

This was a cross-sectional survey study, in which the measurement tool was the French version of the PHEEM.

### Participants

In Rabat, there are 10 university hospitals that provide postgraduate training for 41 specialties, including 4-year residency training for medical specialties and 5-year training for surgical specialties. We included residents from 6 hospitals (Avicenne Hospital, Children’s Hospital of Rabat, Specialties Hospital, Maternity Hospital of Souissi, the National Oncology Institute, and the Dentistry University Center) from the first to the last year of residency over a period of 6 months from January to June 2017. Four hospitals were excluded: Errazi Hospital and Al Ayachi Hospital because they are not located in the city of Rabat, and Maternity Hospital of Orangers and Moulay Youssef Hospital because the residents at those hospitals do not receive obligatory clinical training. The total sample size required to be representative of the residents at hospitals in the city of Rabat for factor analysis of a 40-item instrument was calculated using G*Power (Heinrich-Heine-Universität Düsseldorf, Düsseldorf, Germany; http://www.gpower.hhu.de/). A sample size of 216 residents corresponded to a power of 80% at the 5% significance level when comparing PHEEM scores across 6 specialties using 1-way analysis of variance (ANOVA) to detect a medium effect size (Cohen’s d=0.25).

### Procedure

The questionnaire was distributed by the first author to residents at the hospitals during the study period. All residents in the relevant departments were informed of the study and invited to participate.

### Measure

For use of the French version of the PHEEM [5], permission was received from the translator and the original author [2]. Residents were asked to read each statement carefully and respond to all 40 items using a 5-point Likert scale ranging from ‘strongly disagree’ to ‘strongly agree’ to ‘strongly disagree’ (0, strongly disagree; 1, disagree; 2, uncertain; 3, agree; 4, strongly agree). Four items (numbers 7, 8, 11, and 13) were negative statements that were scored in reverse order. Any items with a mean score of 2 or less should be examined closely, as scores of 2 or lower indicate problem areas [2]. The PHEEM has 3 subscales measuring perceptions of role autonomy (containing 14 items with a maximum score of 56), teaching (containing 15 items with a maximum score of 60), and social support (containing 11 items with a maximum score of 44). Information on age, sex, specialty, residency level, and training hospital were also included as part of the questionnaire. The 40-item PHEEM has a maximum score of 160, which indicates an ideal educational environment as perceived by the respondents [2]. A global score of 0–40 indicates a very poor educational environment; 41–80 indicates plenty of problems; 81–120 indicates more positive than negative, with room for improvement; and 121–160 indicates an excellent environment.

### Statistical analysis

The internal reliability of the overall questionnaire and the 3 subscales was assessed using Cronbach’s α coefficient. By calculating the α coefficient if individual items were deleted, Cronbach’s α was used to identify questions, the exclusion of which would improve the reliability of the tool. We applied factor analysis to investigate the internal structure of the PHEEM and the construct validity of the original 3 subscales. Principal component analysis was used for factor extraction. Varimax rotation was applied and factor loadings above 0.4 were interpreted. Descriptive statistics were used to summarize the survey respondents’ characteristics. Qualitative variables were presented as number and percentages. Quantitative variables were presented as mean±standard deviation for variables with a normal distribution, and as median and interquartile range for variables with skewed distributions. ANOVA was performed to compare total scores, subscale scores, and scores for each item among the residents of the 6 specialties. The threshold for statistical significance was set at P<0.05, and 95% confidence intervals were used. All statistical analyses were performed using SPSS ver. 13.0 for Windows (SPSS Inc., Chicago, IL, USA).

## Results

### Descriptive statistics

**Fig. 1.**
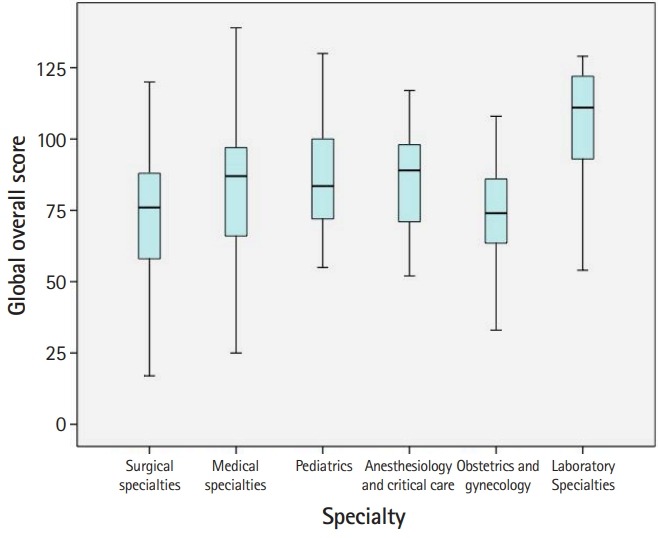
Box plot of overall PHEEM scores in different specialties. PHEEM, Postgraduate Hospital Educational Environment Measure.

In total, 305 questionnaires were distributed. Thirteen residents chose not to participate and 37 questionnaires had missing responses, yielding a response rate of 83.6%. The characteristics of the study respondents are summarized in Table 1. Fig. 1 shows a box plot of the PHEEM overall scores in different specialties. The mean score for each item, each subscale, and the overall PHEEM score are presented in Table 2 (Dataset 1).

### Internal consistency

The 40-item PHEEM questionnaire showed good reliability, with a Cronbach’s α of 0.91. Cronbach’s α values for the 3 subscales of the PHEEM are summarized in Supplement 1.

### Factor analysis

**Fig. 2.**
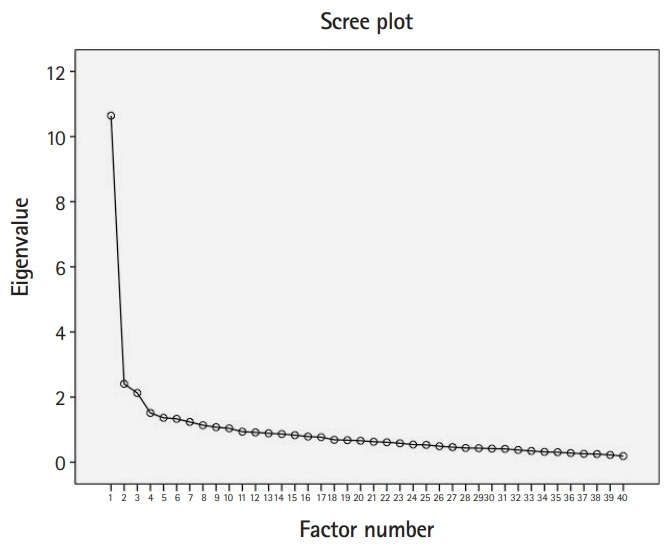
Scree plot of the eigenvalues of the factors’ reliability in principal component analysis.

Principal component analysis of all 40 items (Supplement 2) suggested the presence of 3 or 4 factors, as indicated by inflexion points on the scree plot (Fig. 2), or 10 factors using the criterion of an eigenvalue >1 (accounting for 82.3% of the variance). Varimax rotation identified 35 items (out of 40) that were allocated to 3 factors. Factor 1 explained 30% of the variance and comprised the majority (11 out of 17) of the items that originally belonged to the teaching subscale, 4 items from the autonomy subscale, and 2 items from the social support subscale. Factor 2 explained 10% of the variance and included items from the autonomy subscale (6 out of 14), 5 items from the social support subscale, and 3 items from the teaching subscale. Factor 3 included 2 items from the autonomy subscale and 2 items from the social support subscale and explained 8% of the variance.

### Comparison of residents’ perceptions of their educational environment according to their specialty

We calculated the mean scores for each item, the overall questionnaire, and its subscales, and then compared these scores among 6 groups of residents with different specialties (Table 2). Significant differences were found in the overall and subscale scores among the 6 groups of residents. The residents in laboratory specialties perceived their learning environment more positively than the rest of the residents, particularly the residents in surgical specialties and obstetrics and gynecology. The Moroccan residents in laboratory specialties and anesthesiology and critical care had more positive perceptions of their jobs than the residents of other specialties. The Moroccan residents felt that teaching was moving in the right direction, with a significant difference between the laboratory specialties and the surgical specialties and obstetrics and gynecology. Social support was perceived as “not a pleasant place,” with a significant difference between the pediatrics residents compared to the medical and surgical residents. Table 2 presents the relevant items that accounted for differences among the 6 groups of residents. In the autonomy subscale, the items accounting for the differences were related to the lack of an informative induction program and a handbook for junior doctors, the requirement to perform inappropriate tasks with an inappropriate level of responsibility, and the lack of clear protocols and opportunities to acquire expertise in the appropriate practical procedures. In the teaching subscale, the items accounting for the differences were related to the lack of enthusiastic and organized clinical teachers providing good clinical supervision, feedback, and access to a relevant educational program. In the social support subscale, the items accounting for the differences were related to the presence of racism and sex discrimination, the lack of a “no-blame culture,” inadequate catering facilities and physical safety, failure to obtain ample enjoyment from work, and a lack of good counseling opportunities.

## Discussion

### Key results

In the present study, we evaluated the French version of the PHEEM as a tool to measure the clinical learning environment of Moroccan residents of university hospitals in Rabat. The French version of the PHEEM questionnaire was validated by the present study for the Moroccan learning environment, which is characterized by a high disease burden in a resource-limited country [6]. The PHEEM, which was validated among Moroccan residents recruited from 6 hospitals in this study, has also been used to evaluate the educational environment among residents by other studies in both its original and translated versions [2-5,7]. In the present study, Moroccan residents perceived their educational environment as more positive than negative, but with room for improvement. The PHEEM questionnaire showed high internal consistency, with a Cronbach’s α of 0.91 for all 40 questions, which is similar to the value reported by Roff et al. [2], suggesting that the PHEEM is a suitable multidimensional instrument to measure the educational climate for doctors in training [3]. The PHEEM subscales showed good reliability, especially the education subscale, as has been reported in previous studies [8]. Indeed, confirmatory factor analysis identified 3 factors. The first factor mostly included items from the perception of teaching subscale. The second factor contained a higher proportion of items from the autonomy subscale. The third factor contained items that were correlated poorly with social support. Thus, even if the French version of the PHEEM validated within the Moroccan context is a multidimensional instrument, the PHEEM subscales are not correlated perfectly, especially for social support. A similar finding was reported in a previous study [8].

### Interpretation

The lowest recorded scores were related to the following social support subscale items: “This hospital has good quality accommodation for junior doctors, especially when on call,” “There are adequate catering facilities when I am on call,” and “I feel physically safe within the hospital environment.” These findings highlight the first set of concerns in the Moroccan learning environment, which are related to poor catering and accommodation, phenomena that reflect the lack of efficient management in our context. Moreover, about the half of the residents perceived sex discrimination. This could be explained by the high proportion of female students attending medical schools. Furthermore, more than half of the Moroccan residents felt the lack of good counseling opportunities for junior doctors who fail to complete their training satisfactorily and the absence of a “no-blame culture.” Although the highest scores in the teaching subscale were recorded for the items “My clinical teachers have good teaching skills,” “My clinical teachers are accessible,” and “My clinical teachers encourage me to be an independent learner,” the Moroccan residents perceived some items in this subscale as a problem area, highlighting the second challenge, which is related to the quality of supervision and teaching, particularly among residents of surgical specialties. There is a lack of dedicated educational time, which ultimately defines the role of teachers in practice, affecting a broad range of factors such as the educational program, its organization, learning opportunities, their ability to provide regular feedback, and their feedback on residents’ strengths and weaknesses. Surgical specialties and obstetrics and gynecology have shown lower PHEEM scores among residents in international studies, particularly in the teaching and social support domains. Senior faculty must enhance the quality of supervision and assessment to improve the educational climate of residencies in Morocco, particularly in surgical specialties. The third concern was related to the autonomy subscale, with particular problems including inappropriate tasks and level of responsibility and the lack of information on hours of work, clear clinical protocols, and an informative induction program. In light of the finding of a significant correlation between the educational environment and burnout syndrome among residents in Argentina [9], the above environmental factors may also cause burnout in Moroccan residents. Clear regulations should be applied to define the tasks and responsibility of residents and hours of work, and standardized protocols are required to safeguard the safety of residents and patients. Through this study, we were able to pinpoint specific weaknesses in the educational environment of Moroccan hospitals. However, more specific studies among residents in specific specialties and complementary studies concerning senior faculty members’ perceptions of the learning environment are necessary to identify obstacles that should be explored to create an optimal climate for medical training in Morocco.

### Comparison with previous studies

Moroccan residents perceived their learning environment as more positive than negative, but with room for improvement, like most postgraduate physicians in other countries [10-12]. However residents of surgical specialties and obstetrics and gynecology perceived their educational environment as having “plenty of problems,” especially in the social support and the teaching domains, as reported by similar studies in African and Middle East countries [13-15]. Items related to social support were negatively perceived by Moroccan residents; in particular, the catering facilities, physical safety, and gender discrimination were highlighted as issues to be addressed, as in previous studies in resource-poor and resource-limited countries [10,11,13]. The quality of teaching and supervision has been a source of controversy in the literature, as some studies reported the highest scores for teaching and working together [11], but in most countries, the items on the teaching subscale presented a challenge [13,15]. The deficiencies in the teaching domain reported by the present study included a lack of protected educational time, access to suitable learning opportunities, and constructive feedback and career guidance, and similar challenges have been reported in other international evaluations of the educational climate in both resource-rich and resource-limited countries [11-13]. The nature of the tasks performed, the initial induction program, and clarity of protocols have been identified as challenging issues for improvement in the autonomy subscale, both in Morocco and in other international settings [10-13].

### Limitations

The present study evaluated residents’ perceptions of their educational environment at 6 hospitals, with a high response rate and a representative sample size. However, this study was conducted in a single city, making it difficult to extrapolate these results to the entire Kingdom of Morocco. Therefore, a similar multi-center study is necessary to obtain a broader perspective on the educational environment of Moroccan residents.

### Conclusion

The French version of the PHEEM was confirmed to be a valid and reliable instrument to evaluate the learning environment at university hospital centers in Rabat. Moroccan residents perceived their educational environment as more positive than negative, but with room for improvement. Particularly important challenges included poor infrastructure, inadequate quality of supervision and teaching, and inadequate work regulations.

## Figures and Tables

**Table 1.** Characteristics of participating residents from university hospitals in Rabat, Morocco (N=255)

Characteristic	Value
Age (yr)	29.5±3.2
Sex	
Male	88 (34.5)
Female	167 (65.5)
Residency year	
1	70 (27.5)
2	67 (26.3)
3	48 (18.8)
4	40 (15.7)
5	27 (10.6)
Specialty	
Surgical specialties	69 (27.1)
Medical specialties	70 (27.5)
Pediatrics	61 (23.9)
Anesthesiology and critical care	22 (8.6)
Obstetrics and gynecology	23 (9)
Laboratory specialties	10 (3.7)
Hospital	
Children’s hospital	64 (25.1)
Maternity souissi	45 (17.6)
Avicenne hospital	60 (23.5)
Specialty hospital	43 (16.9)
Oncology national institute	25 (9.8)
Dentistry center	17 (6.7)

Values are presented as mean±standard deviation or number (%).

**Table 2.** Comparison of the mean scores of each PHEEM item across specialties

Domain	Item	Mean (max 4)±SD	Surgical specialties	Medical specialties	Pediatrics	Anesthesiology and critical care	Obstetrics and gynecology	Laboratory specialties	P-value
Autonomy	1. I have a contract of employment that provides information about hours of work.	1.9±1.4	1.7±1.4	1.9±1.3	1.8±1.3	1.8±1.3	1.6±1.4	2.8±1.4	0.4
	4. I had an informative induction program.	1.6±1.4	1.3±1.3	1.6±1.4	1.4±1.2	2.4±1.2	1.1±1	2.8±1.4	**0.002**
	5. I have the appropriate level of responsibility in this post.	2.2±1.2	2±1.4	2.3±1.2	2.2±1.2	1.8±1	2.6±1.1	3.3±1.1	**0.02**
	8. I have to perform inappropriate tasks.	1.6±1.1	1.3±0.9	1.6±1	2.1±1.2	1.8±0.9	1.1±1	1.9±1.1	**<0.0001**
	9. There is an informative junior doctors’ handbook.	2±1.4	2.1±1.5	2±1.1	1.7±1.3	2.8±1.3	1.4±1.3	2.5±1.7	**0.01**
	11. I am bleeped (called) inappropriately.	1.9±1	1.6±0.9	1.8±0.8	2.1±1.2	2±0.8	1.9±0.9	1.9±1.2	0.1
	14. There are clear clinical protocols in this post.	2.2±1.2	2±1.3	2.1±1.1	2.4±1.1	2.5±1.1	1.7±1.2	3.3±0.7	**0.003**
	17. My hours conform to the New Deal .	2.2±1.2	2.1±1.2	2.2±1.1	2.1±1.2	2.2±1.3	1.9±1.1	3.3±1	0.07
	18. I have the opportunity to provide continuity of care.	2.6±1.1	2.5±1.1	2.5±1	2.5±1.1	2.6±1	3±0.8	3.1±1.4	0.1
	29. I feel part of a team working here.	2.7±1	2.5±1.1	2.6±1	2.8±0.7	2.5±0.7	2.7±0.9	2.8±0.7	0.55
	30. I have opportunities to acquire the appropriate practical procedures for my grade.	2.5±1	2.2±1.2	2.2±1	2.7±0.7	2.8±0.8	2.4±0.8	3.1±0.6	**0.006**
	32. My workload in this job is fine.	2±1.2	2±1.3	2±1.1	2.1±1	1.5±1.2	1.6±1.1	2.8±1.3	0.053
	34. The training in this post makes me feel ready to be a specialist.	2.3±1.1	2.2±1.1	2.3±1	2.1±0.9	2.6±0.9	2.1±0.9	2.9±1.3	0.19
	40. My clinical teachers promote an atmosphere of mutual respect.	2.6±1.2	2.4±1.2	2.6±1	2.5±1.1	2.9±0.9	2.5±1.2	2.8±1.4	0.58
	Total subscale score (max=56)	31.9±8.3	31±8.1	32±8.6	33±7.1	34.5±7.8	31±6.4	41.3±10.7	**<0.0001**
Teaching	2. My clinical teachers set clear expectations.	2.2±1.2	2±1.2	2.5±1	2.2±1	2.3±1	1.7±1.3	2.6±1.5	**0.02**
	3. I have protected educational time in this post.	1.7±1.2	1.7±1.3	1.6±1.1	1.7±1	1.3±0.9	1.5±1.2	2.9±1.2	**0.04**
	6. I have good clinical supervision at all times.	2.1±1.2	1.7±1.2	2.2±1	2.2±1.1	1.7±1	2.4±0.8	2.4±0.8	**0.01**
	10. My clinical teachers have good communication skills.	2.5±1.1	2.3±1.3	2.7±0.9	2.4±0.9	2.8±0.8	2.5±1.1	3.1±1.2	0.09
	12. I am able to participate actively in educational events.	2.5±1.1	2.5±1.2	2.5±1.1	2.5±1	2.3±1.1	2.1±1.1	3.5±0.7	0.05
	15. My clinical teachers are enthusiastic.	2.4±1.1	2±1.3	2.5±1	2.5±1	2.4±1	2.4±1.2	3.1±1.2	**0.01**
	21. There is access to an educational program relevant to my needs.	1.4±1.1	1.7±1.1	1.5±1.1	1.2±0.9	1.7±1	1±0.7	1.8±1	**0.04**
	22. I get regular feedback from seniors.	2±1.1	1.8±1	2±1	2.2±1.1	2.3±1	1.8±0.9	2.2±0.9	0.3
	23. My clinical teachers are well organized.	1.2±1.1	1.8±1.2	2.2±1.1	2.5±0.8	2.3±0.9	2±0.9	2.5±1.1	**0.01**
	27. I have enough clinical learning opportunities for my needs.	1.9±1.1	1.7±1.1	2±1.2	2±1	2.5±0.9	1.6±1.1	2.4±1	0.057
	28. My clinical teachers have good teaching skills.	2.8±1	2.6±1.2	2.9±0.9	3±0.7	2.7±0.9	2.4±1	3.5±0.5	**0.008**
	31. My clinical teachers are accessible.	2.8±1	2.6±1.1	2.9±0.9	2.8±0.7	2.6±0.9	2.7±0.9	3±1.3	0.57
	33. Senior staff utilize learning opportunities effectively.	2.3±1.1	2±1.1	2.5±1.1	2.4±1	2.2±0.9	1.8±1	3±1.3	**0.009**
	37. My clinical teachers encourage me to be an independent learner.	2.8±1	2.7±1	2.7±0.9	3.1±0.7	2.4±1.2	2.7±0.9	3.4±0.5	**0.007**
	39. The clinical teachers provide me with good feedback on my strengths and weaknesses.	1.5±1.2	1.6±1.2	1.6±1	0.9±1	1.8±1.1	1.8±1.1	2±1.3	**0.001**
	Total subscale score (max=60)	33.2±10.1	31±11.5	36±9.7	33.9±7.6	34.1±8.7	30±10.1	41.7±11.9	**0.018**
Social support	7. There is racism in this post.	2.1±1.5	1.4±0.9	1.6±1	2.7±1.2	1.6±1.1	1.7±1.1	1.3±0.7	**<0.0001**
	13. There is sex discrimination in this post.	1.8±1.1	1.4±0.9	1.4±0.9	2.7±1.2	1.7±1.2	1.7±1	0.9±0.3	**<0.0001**
	16. I have good collaboration with other doctors in my grade.	3±1	2.9±1.2	3±0.8	3.3±0.8	2.9±0.8	2.8±1	3.2±1	0.3
	19. I have suitable access to careers advice.	1.6±1.2	1.3±1.1	1.6±1.1	1.6±1.2	1.5±1.1	1.6±1.3	2±0.8	0.5
	20. This hospital has good quality accommodation for junior doctors, especially when on call.	0.8±1	0.6±0.9	0.8±1	0.9±0.9	1.3±1	0.6±0.7	1±1.1	0.05
	24. I feel physically safe within the hospital environment.	1.3±1.2	0.9±1.2	1.5±1.3	1.3±1.2	2±1	1±1	1±1	**0.004**
	25. There is a no-blame culture in this post.	1.6±1.1	1.4±1.1	1.4±1.1	1.6±1.1	1.9±0.9	1.6±0.9	2.6±0.9	**0.02**
	26. There are adequate catering facilities when I am on call.	0.6±0.9	0.6±0.9	0.6±0.9	0.5±0.8	1±1.2	0.3±0.7	1.6±1.5	**0.009**
	35. My clinical teachers have good mentoring skills.	2.5±1	2.2±1.1	2.5±1.1	2.7±0.9	2.3±1	2.2±1.2	3±1.1	0.08
	36. I get a lot of enjoyment out of my present job.	2.1±1.1	2±1.1	2±1.1	2.5±1	1.8±0.7	2.1±0.9	2.8±0.6	**0.019**
	38. There are good counseling opportunities for junior doctors who fail to complete their training satisfactorily.	1.2±1.2	0.9±1.1	0.9±1	1.8±1.2	1.4±1.3	1.1±1.2	1.4±1	**0.002**
	Total subscale score (max=44)	18.2±6.1	15.8±5.9	17±6.3	21.5±5.4	19.5±4.9	16.8±4.7	21±5.2	**<0.0001**
Total PHEEM score (max=160)		81.4±21.8	74.3±23.2	82.1±22.1	84.4±18	86.1±18.3	74.5±18	102.2±26.1	**0.089**

Values are presented as mean±SD. PHEEM, Postgraduate Hospital Educational Environment Measure; SD, standard deviation.

## Footnotes

**Data availability** Data files are available from https://doi.org/10.7910/DVN/MSPVKH Dataset 1. Data file and data dictionary.

## Supplementary materials

Data files are available from https://doi.org/10.7910/DVN/MSPVKH

Supplement 1. Mean of PHEEM overall and subscales scores with Cronbach’s α results in each specialty.

Supplement 2. Factor analysis of PHEEM total score.

Supplement 3. Audio recording of the abstract.
