# Supplementary material for: Moroccan residents’ perceptions of the hospital learning environment measured with the French version of the Postgraduate Hospital Educational Environment Measure
Source: J Educ Eval Health Prof. 2020 Jan 31;17:4. doi: 10.3352/jeehp.2020.17.4 (PMC7062605; doi:10.3352/jeehp.2020.17.4)
Supplement: Supplementary file 2 — Supplement 1. Mean of PHEEM overall and subscales scores with Cronbach’s α results in each specialty. [file jeehp-17-04-suppl1.pdf]

Supplement 1. Mean of PHEEM overall and subscales scores with Cronbach's results α in each specialty

Variable	Specialty						Cronbach α	P-value
	Surgical specialties	Medical specialties	Pediatrics	Anesthesiology and critical care	Gynecology-ob-stetrics	Laboratory specialties		
PHEEM total score	74.3 ± 23.2	82 ± 22.1	86.4 ± 18.1	86.1 ± 18.3	74.5 ± 18.1	102.2 ± 26.1	0.91	< 0.0001
Perception education	29.6 ± 11.5	31.9 ± 9.7	33 ± 7.6	34.5 ± 8.7	29.6 ± 10.1	41.3 ± 11.9	0.87	0.007
Perception autonomy	30.5 ± 8.1	34.6 ± 8.6	33.9 ± 7.1	34.1 ± 7.8	30 ± 6.4	41 ± 10.7	0.76	< 0.0001
Perception social support	15.8 ± 5.9	17.3 ± 6.3	21.5 ± 5.4	19.4 ± 4.9	16.8 ± 4.7	21 ± 5.1	0.7	< 0.0001

Values are presented as mean score±standard deviation.
PHEEM, Postgraduate Hospital Educational Environment Measure.

Supplement 2. Factor analysis of the Postgraduate Hospital Educational Environment Measure total score

Original subscale	Item	Autonomy	Teaching	Social support
Autonomy	1. I have a contract of employment that provides information about hours of work.		0.424	
	4. I had an informative induction program.		0.489	
	5. I have the appropriate level of responsibility in this post.		0.511	
	8. I have to perform inappropriate tasks.			0.511
	9. There is an informative Junior Doctors Handbook.			
	11. I am bleeped (called) inappropriately.			0.629
	14. There are clear clinical protocols in this post.		0.409	
	17. My hours conform to the New Deal.		0.421	
	18. I have the opportunity to provide continuity of care.			
	29. I feel part of a team working here.	0.657		
	30. I have opportunities to acquire the appropriate practical procedures for my grade.	0.588		
	32. My workload in this job is fine.		0.466	
	34. The training in this post makes me feel ready to be a specialist.	0.591		
	40. My clinical teachers promote an atmosphere of mutual respect.	0.682		
Teaching	2. My clinical teachers set clear expectations.		0.43	
	3. I have protected educational time in this post.		0.551	
	6. I have good clinical supervision at all times.	0.569		
	10. My clinical teachers have good communication skills.	0.677		
	12. I am able to participate actively in educational events.	0.438		
	15. My clinical teachers are enthusiastic.	0.576		
	21. There is access to an educational program relevant to my needs.		0.643	
	22. I get regular feedback from seniors.			
	23. My clinical teachers are well organized.	0.657		
	27. I have enough clinical learning opportunities for my needs.	0.473		
	28. My clinical teachers have good teaching skills.	0.759		
	31. My clinical teachers are accessible.	0.747		
	33. Senior staff utilize learning opportunities effectively.	0.763		
	37. My clinical teachers encourage me to be an independent learner.	0.586		
	39. The clinical teachers provide me with good feedback on my strengths and weaknesses.	0.422		
Social support	7. There is racism in this post.			0.77
	13. There is sex discrimination in this post.			0.722
	16. I have good collaboration with other doctors in my grade.			
	19. I have suitable access to careers advice.		0.485	
	20. This hospital has good quality accommodation for junior doctors especially when on call.		0.622	
	24. I feel physically safe within the hospital environment.		0.508	
	25. There is a no-blame culture in this post.			
	26. There are adequate catering facilities when I am on call.		0.51	
	35. My clinical teachers have good mentoring skills.	0.67		
	36. I get a lot of enjoyment out of my present job.	0.567		
	38. There are good counseling opportunities for junior doctors who fail to complete their training satisfactorily.		0.439	
