# Supplementary material for: Moroccan residents’ perceptions of the hospital learning environment measured with the French version of the Postgraduate Hospital Educational Environment Measure
Source: J Educ Eval Health Prof. 2020 Jan 31;17:4. doi: 10.3352/jeehp.2020.17.4 (PMC7062605; doi:10.3352/jeehp.2020.17.4)
Supplement: Supplementary file 3 — Supplement 2. Factor analysis of PHEEM total score. [file jeehp-17-04-suppl2.pdf]

Supplement 2. Factor analysis of the Postgraduate Hospital Educational Environment Measure total score

| Original subscale | Item | Autonomy | Teaching | Social support |
|---|---|---|---|---|
| Autonomy | 1. I have a contract of employment that provides information about hours of work. | | 0.424 | |
| | 4. I had an informative induction program. | | 0.489 | |
| | 5. I have the appropriate level of responsibility in this post. | | 0.511 | |
| | 8. I have to perform inappropriate tasks. | | | 0.511 |
| | 9. There is an informative Junior Doctors Handbook. | | | |
| | 11. I am bleeped (called) inappropriately. | | | 0.629 |
| | 14. There are clear clinical protocols in this post. | | 0.409 | |
| | 17. My hours conform to the New Deal. | | 0.421 | |
| | 18. I have the opportunity to provide continuity of care. | | | |
| | 29. I feel part of a team working here. | 0.657 | | |
| | 30. I have opportunities to acquire the appropriate practical procedures for my grade. | 0.588 | | |
| | 32. My workload in this job is fine. | | 0.466 | |
| | 34. The training in this post makes me feel ready to be a specialist. | 0.591 | | |
| | 40. My clinical teachers promote an atmosphere of mutual respect. | 0.682 | | |
| Teaching | 2. My clinical teachers set clear expectations. | | 0.43 | |
| | 3. I have protected educational time in this post. | | 0.551 | |
| | 6. I have good clinical supervision at all times. | 0.569 | | |
| | 10. My clinical teachers have good communication skills. | 0.677 | | |
| | 12. I am able to participate actively in educational events. | 0.438 | | |
| | 15. My clinical teachers are enthusiastic. | 0.576 | | |
| | 21. There is access to an educational program relevant to my needs. | | 0.643 | |
| | 22. I get regular feedback from seniors. | | | |
| | 23. My clinical teachers are well organized. | 0.657 | | |
| | 27. I have enough clinical learning opportunities for my needs. | 0.473 | | |
| | 28. My clinical teachers have good teaching skills. | 0.759 | | |
| | 31. My clinical teachers are accessible. | 0.747 | | |
| | 33. Senior staff utilize learning opportunities effectively. | 0.763 | | |
| | 37. My clinical teachers encourage me to be an independent learner. | 0.586 | | |
| | 39. The clinical teachers provide me with good feedback on my strengths and weaknesses. | 0.422 | | |
| Social support | 7. There is racism in this post. | | | 0.77 |
| | 13. There is sex discrimination in this post. | | | 0.722 |
| | 16. I have good collaboration with other doctors in my grade. | | | |
| | 19. I have suitable access to careers advice. | | 0.485 | |
| | 20. This hospital has good quality accommodation for junior doctors especially when on call. | | 0.622 | |
| | 24. I feel physically safe within the hospital environment. | | 0.508 | |
| | 25. There is a no-blame culture in this post. | | | |
| | 26. There are adequate catering facilities when I am on call. | | 0.51 | |
| | 35. My clinical teachers have good mentoring skills. | 0.67 | | |
| | 36. I get a lot of enjoyment out of my present job. | 0.567 | | |
| | 38. There are good counseling opportunities for junior doctors who fail to complete their training satisfactorily. | | 0.439 | |
